# The Prevalence and Symptoms Characteristic of Functional Constipation Using Rome III Diagnostic Criteria among Tertiary Education Students

**DOI:** 10.1371/journal.pone.0167243

**Published:** 2016-12-20

**Authors:** Ying Jye Lim, Jamaluddin Rosita, Jin Yu Chieng, Abu Saad Hazizi

**Affiliations:** 1 Department of Nutrition & Dietetics, Faculty of Medicine & Health Sciences, Universiti Putra Malaysia, UPM Serdang, Selangor Darul Ehsan, Malaysia; 2 Department of Medicine, Faculty of Medicine & Health Sciences, Universiti Putra Malaysia, UPM Serdang, Selangor Darul Ehsan, Malaysia; Mayo Clinic Arizona, UNITED STATES

## Abstract

**Background and Aims:**

Functional constipation is very common with heterogeneous symptoms that have substantial impact on patient quality of life as well as medical resources which are rarely reported as life-threatening. The aim of this study is to examine the prevalence and symptoms characteristic of functional constipation (FC) by using Rome III diagnostic criteria among tertiary education students with an intention to introduce treatment in the future.

**Methods:**

Demographic, socio-economics characteristics and symptoms of FC using the Rome III criteria were sought using a questionnaire administered to Malaysian students in a tertiary education setting. Other data obtained were the general health status, lifestyle factors and anthropometric measurements. Using a simple random sampling method, a total of 1662 students were recruited in the study with a response rate of 95.0%. Sampled data are presented as frequency and percentage and stratified accordingly into categories for Chi-square analysis.

**Results:**

The prevalence of functional constipation among the students was 16.2%, with a significantly higher prevalence among women (17.4%) than men (12.5%). Hard or lumpy stool, incomplete evacuation, anorectal obstruction and straining were reported as the commonest symptoms experienced. Type 3 was the most frequent stool consistency experienced among the constipated individuals (35.2%). Only 4.4% of individuals reported having less than three defecations per week. Using univariable analysis, FC was significantly associated with sex (odds ratio: 1.48, 95% CI: 1.06–2.06) and age group (odds ratio: 1.34, 95% CI: 1.01–1.79) with *P* value < 0.05 significance level. In multivariate logistic regression analysis, only sex was found significantly associated with FC (adjusted odds ratio: 1.53, 95% CI: 1.08–2.17, *P* < 0.05).

**Conclusions:**

Based on the prevalence rate, constipation is a common problem among tertiary education students (16.2%), with significantly more prevalence among the female respondents. Early detection of symptoms and further intervention studies focusing on treatment recommendation in improving the symptoms are essential.

## Introduction

Functional constipation (FC) is one of the commonest functional gastrointestinal disorders (FGIDs) in the community. It is a worldwide chronic problem with aggravating symptoms but not life-threatening. FC is a condition characterized through bowel symptoms, either primarily or secondarily to an underlying diseases, including the difficulty or infrequent stool passage, hard stool consistency or incomplete stool evacuation [1, 2]. In clinical practise, the physicians regularly defined constipation as an unsatisfying defecations with reduced stool frequency whereas the patients will define their constipation condition based on symptoms such as straining, hard stool, difficulty in defecations and so forth [3–5].

According to Rome III diagnostic criteria for functional constipation, symptoms characteristics were based on abnormal stool type and stool frequency, presence of straining, sensation of incomplete evacuation, anorectal obstruction or blockage and manual maneuvers with occurring at least 25% of defecations. To be defined as functional constipated individuals, the threshold used for respective supportive symptoms should be fulfilled at least often or sometimes of defecations.

The prevalence of FC for the general population in Western countries ranges from 2% to 27%, either through self-reports or Rome criteria definition [6, 7]. In Asia, it affects 15% to 23% among women and about 11% among men, with an increasing trend in the last decade [8]. Study conducted by Suares and Ford [9] showed a pooled prevalence of 11% in the community.

Digestive system disease was the top ten principal causes of morbidity and mortality among Malaysians [10–16] whereby the most frequent incidents reported was colorectal cancer [17]. Constipation was reported to have higher odds towards the occurrence of colon cancer [18]. The morbidity rate of constipation was found to increase with changes in diet, lifestyles, psychological and sociodemographic factors [19]. However, the aetiology of constipation is poorly understood and management has dismal outcome [20].

FC was documented to pose a significant hardship and negatively impact the quality of life [21]. Individuals with constipation were reported to have higher medical utilization and financial costs expenditure [22, 23]. To address this, a study has been conducted to examine the prevalence and symptoms characteristics of FC by using Rome III criteria with the intention to introduce treatment in the future.

## Materials and Methods

### Study setting

A non-experimental, questionnaire-based prospective survey has been conducted cross-sectionally between January 2015 and May 2015 at Universiti Putra Malaysia which is located in the central region of Peninsular Malaysia.

### Sampling method

Simple random sampling method was applied to approach the respondents. This was to obtain the least bias and most generalized feedback [24]. Out of 16 faculties, eight faculties were randomly selected to approach the respondents. The prospective respondents participated in this study were interviewed face-to–face. The respondents were being informed about the detailed research information before consent to participate was obtained. Respondents were then required to fill in the questionnaire by themselves (self-administered) and the collection of questionnaire was done at the same time.

### Subjects

The respondents recruited were Malaysian students enrolled in Universiti Putra Malaysia. The inclusion criteria were students whose age ranged from 18 to 65 years old, both male and female, either from foundation studies, undergraduates or postgraduates, full-time or part-time students. International students (non-Malaysians) or physically disabled students have been excluded during the recruiting process.

### Sample size

By using the established formula by Daniel [25], a total of 1672 respondents recruited in the study will be appropriate. This sample size calculation was done by considered a pooled prevalence of FC among South East Asian of 11.0% [9] at 1.5% accuracy level with 95% confidence interval.

### Ethical consideration

Prior to distribution of questionnaire, this study was approved by the Ethics Committee involving Human Subjects of Universiti Putra Malaysia (JKEUPM) with reference number FPSK(FR14)CT002. All participants have provided verbal informed consent at this screening phase. This is because the respondents were being explained on the research information and contact details were requested for further study when the participants have verbally consented to participate in the research. In addition, each questionnaire had clearly stated that information provided will be used solely for academic research purposes and be kept confidential in the dissemination of the research findings. Thus it was apparent that both ethics committee and participants would recognise that participation in this study is voluntary.

### Instruments

All respondents were required to complete a set of self-administered questionnaire, which consisted of four sections: Part I pertained to general health status, including medical history, record of drug medications, pregnancy and lactation status for women. In Part I, questions such as status of having cardiovascular-related diseases, diabetes, cancer, neurological diseases, bowel disease or other serious illnesses and history of gastrointestinal (GI) surgery were included. This was to identify those participants with alarm symptoms of organic bowel disease and to be ruled out during analysis. Part II was anthropometric measurements which include body weight, body height and body mass index (BMI) classification; Part III consisted of Rome III diagnostic criteria for constipation module and Bristol Stool Chart; Part IV comprised of demographics, socioeconomic status as well as lifestyle factors for example smoking and alcohol consumption habits.

### Rome III Diagnostic Questionnaire for Constipation Module and Bristol Stool Form (BSF) Scale

According to Suares and Ford [9], Rome criteria were developed through symptom-based principle to diagnose various FGIDs, with the initial purpose in assisting patients’ recruitment for clinical researches. In this study, the diagnostic criterion for functional constipation used Rome III Diagnostic Questionnaire for the Adult Functional Gastrointestinal Disorders for Constipation Module (Table 1).

**Table 1:** Rome III diagnostic criteria for functional constipation.

Two or more of the following functional constipation symptoms appear:
- Straining
- Lumpy/hard stools
- Sensation of incomplete evacuation
- Sensation of anorectal blockage/obstruction
- Manual maneuvers to facilitate in defecations
- < Three defecations per week
Loose stool are rarely present without the use of laxative
Insufficient criteria for irritable bowel syndrome (IBS)
Criteria fulfilled for the last three months
Symptomatic onset at least six months prior to diagnosis

The Rome III constipation diagnostic questionnaire response options were on a nominal scale of *yes* or *no*; whereas responses on an ordinal scale of *never or rarely*, *sometimes*, *often*, *most of the time* and *always* for individual frequency thresholds of each question were used to determine the prevalence of symptoms characteristics for functional constipation. The definition of FC requires symptoms to be present at least 25% of defecations. Furthermore, Bristol Stool Chart which is also called Bristol Stool Form (BSF) Scale was used to characterize human stools to seven classifications (Table 2).

**Table 2:** The Bristol Stool Form Scale.

Type	Description
1	Separate hard lumps like nuts (difficult to pass)
2	Sausage shaped but lumpy
3	Like a sausage but with cracks on its surface
4	Like a sausage or snake, smooth and soft
5	Soft blobs with clear-cut edges (passed easily)
6	Fluffy pieces with ragged edges, a mushy stool
7	Watery, no solid pieces, entirely liquid

BSF Scale was used to assess intestinal transit rate by referring to seven types of human stools classification accordingly. Type 1 stool shows the longest time spent in the colon whereas Type 7 indicates the least time spent in the colon. Meanwhile, Type 4 is the most ideal stool type which more closely resembles individuals who defecate once per day and supposed to glide out without any fuss [26].

### Anthropometric measurements

The anthropometric measurements included body weight, body height and body mass index (BMI). The measurements were performed with respondents in barefoot, light clothing and without accessories. The standard weight was measured in kilograms by using an electronic weighing scale (Tanita Cooperation, Tokyo, Japan). Height was measured in the standing position by using a stadiometer (SECA 206 microtoise tape, SECA Corporation, Hamburg, Germany). Respondents were required to stand straight feet together, arms hanging loosely by sides, face forward and knee straights, heels, buttocks and shoulder blades in contact with the vertical surface of the wall. The measurement of body weight and height were taken twice for the average value, to the nearest 0.5 kg and 0.1 cm, respectively.

BMI was calculated as weight in kilograms divided by square of the height in meters (kg/m^2^). The WHO [27] classification for BMI cut-offs was used as followings: underweight (BMI < 18.5 kg/m^2^); normal body weight (18.5 ≤ BMI < 25.0 kg/m^2^); overweight (25.0 ≤ BMI < 30.0 kg/m^2^); and obese (BMI ≥ 30.0 kg/m^2^).

### Statistical analysis

The sampled respondents were interviewed and the data were coded for analysis and data handling. The data was stratified accordingly into categories of age (18–24 years and ≥25 years) according to previous studies carried out among Malaysian and university students [28–30], ethnicity of either Malay or non-Malay, academic programme (which was classified into three levels: foundation studies, undergraduates and postgraduates) and smoking habits (current smoker or non-current smoker). Descriptive statistics, comprising of proportions and percentages were presented. In addition, univariable and multivariable logistic regression analysis were conducted to examine the association of functional constipation with the predictors including sex, age, ethnicity, academic programme, marital status, income, smoking status, alcohol intake and body weight status. Both results were presented as crude odds ratios (ORs) and adjusted ORs respectively with 95% confidence intervals (CIs). The statistically significance level was specified at *P*-value of 0.05. All analysis was conducted by using SPSS version 22 (SPSS Inc., Chicago, IL, USA).

## Results

Of the 1750 questionnaires distributed, 1662 sets were completed and analysed, with a questionnaire response rate of 95.0%. As for the characteristics (Table 3), the respondents comprised of 1262 (75.9%) women and 400 (24.1%) men. The mean age of the study participants was 23.3 ± 2.8 years, with an age range of 18 to 53 years old. Majority of the respondents were Malay students (72.3%), doing their undergraduates studies (80.6%), neither taking any tobacco (98.1%) nor an alcohol drinker (92.4%). Out of the 1662 students, 13.1% of them were classified as overweight and 5.4% were obese, whereas 13.8% were underweight. Table 4 displays the general health status of the respondents. Less than three percent of them were having cardiovascular-related diseases, diabetes, cancer, neurological diseases, or other serious illnesses, undergone GI surgery or on GI medication. Only one percent was reported on regular laxative use (more than once in a week).

**Table 3:** Demographic, socioeconomics characteristics, lifestyle factors and body weight status of the respondents.

Variables	Categories	Respondents	
		n	%
**Sex**	Male	400	24.1
	Female	1262	75.9
**Age (years)**	18–24	1244	74.9
	25–34	396	23.8
	35–44	20	1.2
	45–54	2	0.2
**Ethnicity**	Malay	1203	72.3
	Chinese	327	19.7
	Indian	68	4.1
	Others	64	3.9
**Academic programme**	Foundation	201	12.1
	Undergraduates	1339	80.6
	Postgraduates	122	7.3
**Marital status**	Single	1600	96.3
	Married	58	3.5
	Divorced	4	0.2
**Household income**	Less than RM1000	325	19.6
	RM1001 –RM3000	688	41.4
	RM3001 or more	649	39.0
**Lifestyle factors**			
**Smoking status**	Current smoker	31	1.9
	Non-current smoker	1631	98.1
**Alcohol consumption**	Yes	127	7.6
	No	1535	92.4
**Body weight status**	Underweight (< 18.5 kg/m^2^)	230	13.8
	Normal weight (18.5–24.9 kg/m^2^)	1124	67.6
	Overweight (25.0–29.9 kg/m^2^)	218	13.1
	Obesity (> 30.0 kg/m^2^)	90	5.4

RM: Ringgit Malaysia.

**Table 4:** General health status of the respondents.

Variables	Yes	No
Disease/serious illnesses	46 (2.8)	1616 (97.2)
GI surgery	35 (2.1)	1627 (97.9)
GI medication	41 (2.5)	1621 (97.5)
On regular medication	54 (3.3)	1608 (96.75)
Probiotic product	84 (5.1)	1578 (94.9)
Laxative	16 (1.0)	1646 (99.0)
Pregnancy (n = 1262)	16 (1.3)	1246 (98.7)
Lactation (n = 1262)	14 (1.1)	1248 (98.9)

### Prevalence and symptoms characteristic of functional constipation

**Fig 1:**
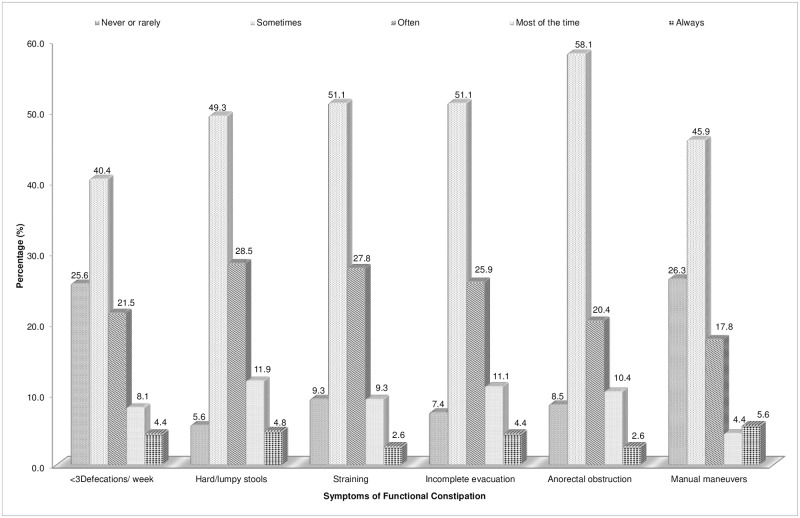
Percentage of FC symptoms characteristic among constipated individuals (n = 270).

The prevalence of functional constipation among UPM students was 16.2% with 17.4% among women and 12.5% among men, according to Rome III diagnostic criteria for constipation and excluding those with alarm symptoms of organic bowel diseases. Of 270 functional constipated individuals, the symptoms characteristic of functional constipation was investigated. Hard or lumpy stool, incomplete evacuation, anorectal obstruction and straining were found the most common symptoms experienced (Fig 1). It was reported that 90.7% to 94.4% of the constipated individuals encountered at least sometimes of the respective symptoms.

Among the constipated individuals, 74.4% of them were reported to have less than three defecations in a week. There were 4.4% of them who always experienced only zero to two times of bowel movement per week. Hard or lumpy stools were the most common constipation symptom encountered among the constipated individuals. Majority of them (94.4%) fulfilled the symptoms as functional constipation criteria. There were 90.7% of the constipated individuals showed the need to strain or encountered difficulty during their stool evacuation. Among the 270 constipated individuals, 92.6% of them were reported with the sensation of incomplete emptying experienced during their bowel movement. As for sensation of anorectal obstruction or blockage, there was 91.5% manifested the feeling of anal obstruction. There was 73.7% of the individuals indicated the need to manual manuevers to facilitate the defecation process.

As for the stool type characterised using BSF scale (Table 5), Type 3 was the commonest experienced among the constipated individuals (32.2%). Meanwhile, 2.6% and 18.9% of constipated individuals were self-reported to have Type 1 and Type 2 stools, respectively. There were 6.6% of constipated individuals presented to have inconsistent stool type.

**Table 5:** BSF Scale of the constipated individuals (n = 270).

Type	Description	n (%)
1	Separate hard lumps like nuts (difficult to pass)	7 (2.6)
2	Sausage shaped but lumpy	51 (18.9)
3	Like a sausage but with cracks on its surface	95 (35.2)
4	Like a sausage or snake, smooth and soft	52 (19.3)
5	Soft blobs with clear-cut edges (passed easily)	25 (9.3)
6	Fluffy pieces with ragged edges, a mushy stool	20 (7.4)
7	Watery, no solid pieces, entirely liquid	2 (0.7)

### Univariable and multivariable analysis of FC according to demographic, socioeconomics characteristics, lifestyle factors and body weight status

The association of independent variables with FC was displayed in Table 6. For univariable logistic regression analysis, significant association was reported between FC with sex (OR: 1.48, 95% CI: 1.06–2.06) and age group (OR: 1.34, 95% CI: 1.01–1.79) with *P* value < 0.05 significant. Female was found to be more prevalent of having FC based on Rome III diagnostic criteria. In addition, older age group has more tendencies toward FC. However, from the findings, there was no significant association between FC with ethnicity, academic programme, marital status, income, smoking status, alcohol consumption and body weight status. With multivariable logistic regression analysis, only sex variable was found associated with FC (adjusted OR: 1.53, 95% CI: 1.08–2.17, *P* < 0.05).

**Table 6:** Univariable and multivariable analysis of functional constipation using Rome III criteria by demographic, socioeconomics characteristics, lifestyle factors and body weight status.

Parameters	FC, n (%) (n = 270)	No FC, n (%) (n = 1392)	Crude OR (95% CI)	Adjusted OR^1^ (95% CI)	*P* value^1^
**Sex**					
Male	50 (12.5)	350 (87.5)	1.00	1.00	
Female	220(17.4)	1042 (82.6)	1.48 (1.06, 2.06)^2^	1.53 (1.08, 2.17)	0.02^1^
**Age group**					
18–24 years	188 (15.2)	1055 (84.8)	1.00	1.00	
≥ 25 years	81 (19.4)	337 (80.6)	1.34 (1.01, 1.79)^2^	1.29 (0.92, 1.80)	0.15
**Ethnicity**					
Malay	188 (15.6)	1015 (84.4)	1.00	1.00	
Non-Malay	82 (17.9)	377 (82.1)	1.17 (0.88, 1.56)	1.13 (0.82, 1.56)	0.46
**Academic programme**					
Foundation	30 (14.9)	171 (85.1)	1.00	1.00	
Undergraduates	214 (16.0)	1125 (84.0)	1.08 (0.72, 1.64)	1.04 (0.68, 1.57)	0.87
Postgraduates	26 (21.3)	96 (78.7)	1.54 (0.86, 2.76)	1.18 (0.62, 2.24)	0.61
**Marital status**					
Single/Divorced	258 (15.2)	1346 (84.8)	1.00	1.00	
Married	12 (19.4)	46 (80.6)	1.36 (0.71, 2.61)	1.18 (0.59, 2.37)	0.63
**Household income**					
≤ RM3000	164 (16.2)	849 (83.8)	1.00	1.00	
> RM3000	106 (16.3)	543 (83.7)	1.01 (0.77, 1.32)	0.99 (0.76, 1.30)	0.94
**Smoking status**					
Current smoker	5 (16.1)	26 (83.9)	0.99 (0.78, 2.61)	1.31 (0.48, 3.60)	0.60
Non-current smoker	265 (16.2)	1366 (83.8)	1.00	1.00	
**Alcohol consumption**					
Yes	24 (18.9)	103 (81.1)	1.22 (0.77, 1.94)	1.14 (0.67, 1.92)	0.64
No	246 (16.0)	1289 (84.0)	1.00	1.00	
**Body weight status**					
Underweight	46 (20.0)	184 (80.0)	1.00	1.00	
Normal weight	177 (15.7)	947 (84.3)	0.75 (0.52, 1.07)	0.74 (0.51, 1.06)	0.10
Overweight & obesity	47 (15.3)	261 (84.7)	0.72 (0.46, 1.13)	0.72 (0.46, 1.15)	0.17

^1^Adjusted for all parameters listed in Table 1, *P* < 0.05 significant

^2^Significant on univariate analysis, *P* < 0.05 significant

OR: Odds ratio; CI: Confidence interval; RM: Ringgit Malaysia

## Discussion

Constipation is affected by many factors. Functional constipation is defined when there are no anatomical or physiological causing factors. It was reported as a common intestinal disorders found prevalent in elderly and middle-aged women yet younger individuals nowadays were shown with constipation due to changes in sociocultural, diet, lack of physical activity and stress factors [31, 32]. This survey was conducted to estimate the prevalence of FC among the students from tertiary education setting by using Rome III criteria. The study has identified the prevalence of functional constipation among the students was consistent with the prevalence rate reported by previous studies.

In general population of the Western countries, the prevalence stated was ranged from 2% to 27% [6, 7]. The prevalence of functional constipation among Asian was up to 14.3% [33–36] whereas it was reported that the pooled prevalence of functional constipation was 11.0% among South East Asian, extracted from a total of 11 studies [9]. To our knowledge, the prevalence rate of functional constipation in Malaysia has not been revealed. This has been highlighted in a local clinical study report using probiotics dairy drinks for constipation [37]. Even though there were several studies on the prevalence of irritable bowel syndrome (IBS), specifically constipation-predominant IBS has been published in Malaysia [38–40]; however, to the best of our knowledge, there is no published study about the reported prevalence rate of functional constipation among Malaysians.

As for constipation symptoms characteristics, the individuals were demonstrated to more frequently experience the difficulty during stool passage and consistency rather than defecation frequencies. The results corroborated with the findings illustrated by Wald et al. [4] whereby individuals more often define constipation as symptoms of straining, hard stool, discomfort, difficulty in passage; whereas clinical definition was primarily based on infrequent stool frequency [3]. Another earlier study also revealed that defecation frequency alone could not generalise the condition of having constipation as there were several other criteria disclosed as symptoms of constipation [5]. A possible explanation for this condition is individuals who are having normal frequency of bowel movement might experience other constipation symptoms, include hard or lumpy stool, incomplete evacuation, straining during defecations and so forth, which has been similarly reported by Dong et al. [32]. This has further supports from our finding where Type 3 stool was listed as the commonest pattern. This could be due to the consistency of Type 3 stool as typical for latent constipation which will requires straining during the defecations process [26]. Recent study conducted by Chu and Hou [41] revealed that Chinese physicians were having different point of view on the stool types correspondence with bowel movement condition. Majority of them categorised Type 3 as constipated in addition to Type 1 and Type 2 [41].

On the prevalence of FC among sex variable, this study illustrated that women have higher prevalence than men. The present finding is in agreement with the outcomes of other researches which found that FC was more prevalent among female [5, 9, 21, 42–44]. In Asia, the prevalence of FC was 15% to 23% among women and about 11% among men, with an increasing trend in the last decade [8]. According to the report of WGO [2], the occurrence of constipation stems from factors such as dietary pattern, fluid intake and psychological factor. Yet, for the observed higher prevalence among women could be attributed by hormonal changes, eating behaviour and history of physical as well as emotional problems [45, 46]. Increased progesterone hormone in women will caused the changes in muscle tone and motility in the gastrointestinal tract to slow down, which resulted in the occurrence of constipation [47]. Moreover, emotional status was reported with increased in prevalence of constipation [48].

In current study, comparing age with the prevalence of FC as resulted from univariable analysis showed that there was a significant association between these variables. This study confirmed that constipation was reported to increase with age, which also accorded with previous observations [5, 9, 43, 49–51]. As individuals aged, they might have difficulty in defecation. This could be due to age-related changes in anorectal physiology and gradual colonic dysfunction, impaired rectal sensation and low calories intake that caused reduction in stool volume and bulk [5, 51]. Therefore, careful attention to diet and exercise need to be looked after in order to prevent an impaction on the bowel problem from recurring. Moreover, there was a study that indicated FC will arise from physical inactivity, even among healthy individuals [52].

As for other socioeconomic factors, no significance differences were found between ethnicity, academic programme, marital status and income with the prevalence of constipation. However, the findings of the current study do not support the previous research, which reported that lower socioeconomic status and education level were found to be associated with constipation [3, 4, 36, 42, 53, 54]. Furthermore, Suares and Ford [9] indicated that lower socioeconomic status has an impact in increasing the prevalence of functional constipation. For educational level, there were some contradictions on the research outcomes. There were studies shown that highly educated individuals have higher constipation disorders [31, 55] whereas no reported relationship was found of educational level with the occurrence of constipation [56]. These rather contradictory results may be due to different sampling method, definition used for FC, data collected either through self-report by the respondents or Rome criteria [57].

In the meantime, our study showed that the prevalence of constipation has no significant relationship with lifestyle factors and body weight status. There are similarities in the findings between the body mass index classification and smoking status with the prevalence of constipation in this study and those described by previous studies [21, 58]. In contrast to earlier findings for alcohol intake, there was no significant association linked in this study because of the very small number of drinkers in current study. Reports from past studies showed that quantity of alcohol intake was found to affect intestinal transit time [59, 60].

To update, Rome IV criteria have superseded Rome III criteria in May 2016. New diagnoses have been included as updated criteria in Rome IV which are reflux hypersensitivity syndrome, cannabinoid hyperemesis syndrome, chronic nausea/vomiting syndrome caused by narcotic bowel syndrome and lastly opioid-induced constipation (OIC) has been added in supplementary to types of bowel disorders [61]. For functional constipation in Rome IV, presence of abdominal pain and/or bloating had been recognized at finite threshold [62] and the duration or frequency criteria for the supportive symptoms were amended as compared with the previous Rome III criteria which has been discussed earlier [63].

Meanwhile, the Rome IV criteria revised the rating to a 0–10 numeric scale of severity with *0* indicate *0% (never)* to *10* as *100% (always)*. The threshold for each symptom has been set accordingly, which are fewer than three spontaneous stools per week and sensation of anorectal blockage (at least 40% of stools), straining and incomplete emptying (at least 50% of stools), manual maneuvers (at least 20% of stools) and lumpy or hard stools (at least 50% of stools). In Rome IV, Bristol Stool Chart has been adopted in the criteria to identify abnormal stool type [61]. To be more specific, Type 1 or 2 stool need to present at least 50% of defecations, including when not on medications for diarrhea; whereas Type 6 or 7 stool encountered for 20% of less of the bowel movements when not on constipation medications or therapy [61]. Similar to Rome III, the respective individual needs to fulfil at least two or more of the aforementioned supportive symptoms. In Rome IV, to be diagnosed as FC, individual should not on opioids drugs for treating pain in OIC or encounter abdominal pain which at a frequency sufficient to meet criteria for IBS, respectively [62]. The required duration of six months or more of symptoms onset is to be considered as one of the diagnostic criteria [61]. Due to more definite criteria had been set in Rome IV, we therefore postulated that the prevalence of functional constipation could be lower.

## Contributions and Limitations of the Study

To our knowledge, this is the only empirical evidence study on FC among Malaysians especially in tertiary education. This research was aimed to identify the prevalence and symptoms characteristics of FC among students in tertiary education setting. Furthermore, the epidemiological characteristics of FC which revealed in this study will perhaps enable the development of public policies to advocate an increase in the awareness and early diagnosis of these symptoms. This is because through an early detection of symptoms and to seek treatment among respective individuals could as well prevent from complications of the chronic condition.

Moreover, this contributes in obtaining a better quality of life, especially among female and elderly population. On the other hand, there were limitations worth noting in this study. Physical activity level, emotional and psychological factors, dietary intake as well as the level of quality of life should take into account for future study in order to correlate the contributing factors toward the occurrence of symptoms. However, this study is the screening phase to look at the prevalence of functional constipation among the students. All the variables aforementioned were being measured in the second part of the research project which is going to be published later. In addition, only university students were involved in this study, which was not representing all adults in Malaysia.

## Conclusion

In conclusion, constipation is a common problem among the tertiary education students. Our study showed that the prevalence of FC among the students was 16.2%, with significantly higher prevalence among female (17.4%). Even though constipation does not cause mortality, however it is demanding on medical costs and will sequelae the condition if remain untreated. The pathogenesis of constipation is complicated [20] and thus, early detection of symptoms is critical for future focus on treatment recommendation and prevention of complications demonstrates benefits on improving the quality of life of the respective individuals.

## Supporting Information

S1 File Screening Data—Prevalence of FC among Tertiary Education Students. Minimal data set used to reach the conclusions drawn in the manuscript.
(XLSX)
